# Biostatisticians Meet AI: Navigating Shifts While Preserving Principles

**DOI:** 10.1002/sim.70271

**Published:** 2025-09-22

**Authors:** Bin Zhu

**Affiliations:** ^1^ Division of Cancer Epidemiology and Genetics National Cancer Institute Bethesda Maryland USA

**Keywords:** artificial intelligence, benchmark, biostatistics, ChatGPT, large language model

The manuscript *ChatGPT as a Tool for Biostatisticians: A Tutorial on Applications, Opportunities, and Limitations* by Dobler et al. provides a timely and insightful assessment of how large language models (LLMs) can assist in biostatistical work. The authors showcase several real‐world use cases to illustrate ChatGPT's capabilities and pitfalls in our domain. This breadth of examples, each repeated in multiple ChatGPT sessions to assess consistency, offers a nuanced picture of what current LLMs can and cannot do. The effort is commendable for moving beyond abstract discussions and demonstrating concrete applications of ChatGPT in biostatistical workflows. Notably, the tutorial highlights instances of impressive performance (e.g., successful latent class analysis) alongside clear failures (e.g., incorrect meta‐analyses), thereby educating biostatisticians on both the opportunities and limitations. The authors' ultimate message is one of cautious optimism: LLMs can streamline routine tasks for biostatisticians, but their outputs must be verified by a knowledgeable user. By openly sharing both positive results and errors, Dobler et al. set a pragmatic tone and reinforce that LLMs, while transformative, are no substitute for sound statistical judgment. We applaud the authors for this balanced, real‐world tutorial, which serves as a starting point for biostatisticians interested in harnessing artificial intelligence (AI) in practice.

## 1 From Calculator to LLMs: Shifting Productivity Paradigms

The way we conduct statistical analysis has continually evolved with technology. Not long ago, biostatisticians produced ANOVA tables using a calculator and retrieved *p* values from printed tables. The computer revolution transformed the discipline by allowing biostatisticians to write, share, and reproduce entire analytical workflows using R or Python.

Now, we stand before another paradigm shift: the rise of reasoning LLMs such as ChatGPT o1 and deepseek‐R1. Unlike traditional statistical software which executes user‐written codes, and unlike earlier foundation LLMs that produce a quick answer after a brief forward pass, reasoning LLMs devote substantial inference‐time compute to reasoning. This means that given a hard question, reasoning LLMs can search the internet, extract related information, explore numerous solution paths, score them, and iteratively refine their output over minutes (or longer) before committing to a final answer. As a result, we are beginning to witness reasoning LLMs solving challenges once deemed too hard for AI. For example, Google DeepMind's *Gemini Deep Think* has recently won a gold medal in the 2025 International Math Olympiad (IMO) by solving five of six problems. It suggests that an AI agent could tackle analytical tasks that we traditionally assumed required a human expert. Biostatisticians therefore find themselves equipped with a fundamentally new kind of tool that not only crunches numbers but can also plan, synthesize, reason, and prove in ways that echo human problem‐solving. Of course, such a tool must be used carefully (a point we return to later), but there is no doubt that reasoning LLMs are poised to redefine our workflow.

History shows that when we adopt new tools, our productivity can leap. Those who first embraced programmable software quickly outpaced those sticking to calculators; in the same vein, those who learn to effectively use AI may find they accomplish in hours what used to take days. The challenge for all of us is to adapt and integrate these new tools thoughtfully into our workflow.

## 2 Benchmarking AI on Biostatistical Challenges

To harness these opportunities, we need to measure and decide what LLMs can do in our domain. Building on the example spearheaded by Dobler et al., we advocate the creation of a comprehensive, carefully curated benchmark, a “biostatistics qualifying exam” for LLMs. Can ChatGPT pass a typical PhD qualifying exam in biostatistics? How does it perform on problems from survival analysis, longitudinal analysis, or clinical trial design? Establishing a standard evaluation set, perhaps drawn from recent exam questions or real‐world collaboration projects, would allow the community to track LLMs' performance across iterations and identify strengths and weaknesses specific for biostatistics. Other fields have started doing this: For example, ChatGPT has been evaluated on the United States Medical Licensing Examination (USMLE) [1] and on the Uniform Bar Examination (UBE) [2]. In chemistry, ChemBench was created as a 2700‐item benchmark of chemical knowledge and reasoning [3]. Biostatistics should be no exception. A biostatistics “arena” for LLMs would quantify LLMs' strengths and expose their domain‐specific weaknesses (e.g., do LLMs struggle more with survival analysis theory or with experimental design requests?). Such an initiative could tailor improvements for biostatistics and guide us in knowing when to rely on the LLMs. Such a benchmark would function as a *biostatistics Turing test*: We challenge LLMs with our hardest questions and measure how closely LLMs approach expert‐level performance. The results would be both humbling and motivating, reminding us that what we find hard, LLMs might eventually handle, and what they still get wrong, we must carefully safeguard.

## 3 AI Agent Demands Rigor, Not Relaxation

It might be tempting to view LLMs as an “autopilot” for analyses, but the opposite is true. The rise of LLMs demands more statistical rigor and critical thinking from us, not less. Why? First, LLMs are not infallible, as shown by Dobler et al. and another report [4]. They may produce output that is perfectly fluent and convincing, yet subtly wrong or based on faulty reasoning. Second, effective use of LLMs requires critical thinking in the guidance we provide. LLMs often need precisely crafted prompts and follow‐up questions to produce correct outputs. The aforementioned tutorial paper demonstrates this well: ChatGPT‐4o could eventually solve some biostatistics problems but only with careful guidance and multiple attempts. In practice, that means a biostatistician must know when the LLMs' answer is wrong, how to steer it in the right direction, and how to break a complex task into manageable sub‐tasks for LLMs with clear instructions. As we integrate these tools, maintaining a skeptical, analytical mindset is paramount—the last thing we should do is turn our brains off and trust every answer from LLMs.

## 4 Redefining the Biostatistician's Role in the AI Era

All these developments beg the question: What does it mean to be a biostatistician today? In the age of AI, our identity and value proposition are inevitably evolving. We believe the essence of our profession remains the same: to search for truth in data with scientific rigor, but how we fulfill that mission is shifting. The biostatistician of tomorrow might spend less time on tedious coding and more time on problem solving: formulating the right questions, devising rigorous analysis plans, and ensuring conclusions are backed by sound evidence. Our role becomes director and critic of AI. We will be the ones ensuring that study designs make sense, that assumptions are checked, and that ethical considerations are accounted for. In short, we guard the validity of the scientific process.

In this sense, we might compare the AI revolution to the advent of calculators or statistical software: those tools didn't render mathematicians or statisticians obsolete; instead, they freed them from repetitive calculation to focus on insight and discovery. Likewise, biostatisticians armed with AI are not diminished; they're augmented. They can venture into new fields more readily, as the AI can help summarize unfamiliar literature or suggest analytical approaches from other domains. They can communicate more effectively, using the AI to draft lay explanations or visualizations for interdisciplinary teams. Perhaps our collaborations will deepen, as we're able to bridge gaps in understanding faster. We provide the careful thinking, the statistical expertise, and the skeptical eye that ensure AI‐driven analyses are correct and meaningful. We ask the questions that the AI doesn't know to ask. We interpret its outputs against real‐world complexities and decide an actionable plan.

Embracing AI should motivate us to reaffirm the distinctive strengths of our profession. The tools will change, from calculators to R, and now to LLMs, but our core principles do not. It is our responsibility to equip ourselves and future biostatisticians with AI fundamentals, effective prompt skills, and rigorous methods to evaluate AI outputs. At the same time, we must safeguard our identity as scientists devoted to uncovering truth in data. In the age of AI, biostatisticians' value will lie not in how fast they can crunch numbers but in how wisely they can harness these new powers and how steadfastly they can ensure that statistical rigor and critical thinking remain the bedrock of all we do.

In conclusion, the advent of AI is transforming biostatistics, opening new frontiers of efficiency and capability. We should lean into this transformation with optimism and curiosity, much as the authors of the tutorial have while carrying forward the timeless principles of our field. The future of biostatistics in an AI‐augmented era is bright, so long as we embrace the new technology with eyes open, minds engaged, and the rigor of our discipline held firmly intact.

## Conflicts of Interest

The author declares no conflicts of interest.

## Data Availability

Data sharing not applicable to this article as no datasets were generated or analyzed during the current study.
